# Self-rated quality of life of city-dwelling elderly people benefitting from social help: results of a cross-sectional study

**DOI:** 10.1186/1477-7525-11-181

**Published:** 2013-10-29

**Authors:** Marek Bryła, Monika Burzyńska, Irena Maniecka-Bryła

**Affiliations:** 1 Department of Social Medicine, the Chair of Social and Preventive Medicine of the Medical University of Lodz, ul. Żeligowskiego 7/9, Łódź 90-752, Poland; 2 Department of Epidemiology and Biostatistics, the Chair of Social and Preventive Medicine of the Medical University of Lodz, ul. Żeligowskiego 7/9, Łódź 90-752, Poland

**Keywords:** Ageing population, Social help, Quality of life, Poland

## Abstract

**Background:**

The percentage of people aged 65 or older living in Poland is 13.6%, but 17.2% in Łódź. The aim of the study was to identify factors correlating with the self-rated quality of life of elderly inhabitants of cities applying for social help, on the basis of a cross-sectional study.

**Methods:**

The study was conducted in Łódź, a large Polish city, between September 2011 and February 2012 in a group of people applying for help in the Municipal Social Welfare Centre. Four hundred and sixty-six respondents aged 65 or older were included in the study. The tool used in the study was an interview questionnaire. The respondents answered questions on their demographic situation, living conditions, financial, health and social situation. The authors also applied the WHOQOL-BREF Questionnaire, the Activities of Daily Living Scale (ADL) and the Geriatric Depression Scale (GSOD). For statistical purposes, the authors used single- and multiple-factor regression and the Statistica 9.0 Program. The results were presented as an odds ratio (OR) with a 95% confidence interval (CI); the adopted significance level was p < 0.05. The authors applied the Pearson’s *x*^*2*^ test in order to evaluate the structure of the studied group and the subpopulation, who were aged 65 or older and using social help, throughout the city.

**Results:**

Logistic regression confirmed that a high quality of life depends on the following variables: university education (OR = 2.31; p < 0.05), an income which is sufficient to live (OR = 1.63; p < 0.05), no heart palpitations (OR = 2.32; p < 0.05), stable blood pressure (OR = 2.32; p < 0.05), no headaches (OR = 1.55; p < 0.05), no pain in the chest (OR = 1.51; p < 0.01), no shortness of breath (OR = 1.51; p < 0.01), no tiredness (OR = 2.08; p < 0.05), a score on the Geriatric Depression Scale pointing to a lack of suspected depression (OR = 9.88; p < 0.001 if the person does not suffer from depression and OR = 6.33; p < 0.001 if there is uncertain depression) as well as not using nursing services, a score on the ADL Scale confirming the person’s fitness and participation in family gatherings.

**Conclusions:**

A subjective evaluation of the quality of life of the elderly depends on many factors. An identification of these factors might be helpful in implementing steps aimed at improving the quality of life of elderly people who, as a consequence, will need less social help: particularly nursing services.

## Introduction

The concept “QOL – quality of life” appeared as scientific terminology in the 1960s [1,2]. It is described as “the degree to which persons perceive themselves able to function physically, emotionally, mentally, and socially. In a more quantitative sense, an estimate of remaining life free of impairment, disability, or handicap, as use in the expression Quality-Adjusted Life Years” [3]. In medical sciences the problem of a quality of life started to become a focus of scientific interest in the 1970s. Studies conducted in that period concentrated on health and non-health issues which were observed in respondents who had been affected by various diseases. The quality of life was also connected with medical and non-medical health care. The man who introduced the notion “quality of life” to medical research studies was D. Karnofsky. He paid particular attention to the subjective situation of the patient [4]. In Poland the issue of the quality of life started to be discussed in the 1980s. In this period of time a lot of changes occurred. Professionals started to notice the importance of psychological factors in the medical treatment process, promoting health and a healthy lifestyle. In the medical and psychological sciences, attempts were made to define a standard concept of the quality of life. The World Health Organization (WHO) attempted to move this notion from social sciences to medical sciences and described it as: “subjective perception of the person’s position in life in the particular culture and a system of values in which the person lives as well as his/her position and expectations, objectives, and norms that lie ahead of the person”. The definition of health, adopted in 1948 by the WHO gave an impulse for further considerations. According to the organization health should not be measured only with negative features such as mortality or morbidity; it should be evaluated in a wider bio-psycho-social context because “*it is a state of complete physical, mental and social well-being and not merely the absence of disease or infirmity”*[5]. Thus, it can be concluded that a disease is not only a physical indisposition of the person but it also relates to the person’s mental, social and spiritual spheres. Thus, it was necessary to introduce a measure of mental and social well-being. Theoretical models of the quality of life which depended on health were designed, Health Related Quality of Life – HRQOL [6]–[11]; these assume that health is one of the most important factors which affects the quality of our life [12]–[19].

The increase in the elderly population in a period of the demographic and socio-economic transformation is the reason for conducting empirical studies on the quality of life related to health and socio-economic situation. It should be pointed out that the authors carried out their own study in Łódź, which is inhabited by 742 000 people, and whose population is characterized by many negative epidemiological measures related to health, as well as the highest ageing rate in Poland: the percentage of people aged 65 years or older is 17.2%, whereas in the whole country it is 13.6%. The number of nursing and medical care services available for this age group is rapidly growing. It is worth mentioning that the average number of expected lifespan for inhabitants of Łódź in 2011 was 70.1 for males and 78.4 for females. These values were lower than the average value for Poland by 1.9 years for males and 2.6 years for females, and also lower than average life span for inhabitants of the European Union by 6.0 and 3.8 years respectively [20,21].

Łódź, being a typical industrial city, had to confront serious problems after the year 1989, i.e. a period of social and economic change and the introduction of mechanisms of market economy [22]. The decline of the textile industry negatively affected the situation of inhabitants and contributed to growing unemployment. This phenomenon particularly affected people at production age. However, it also negatively affected inhabitants at post-productive age. For the first time they did not have a chance to earn extra money but they had to live on low, often insufficient pensions. This also led to unemployment in the closest family members. Their financial help for the family members often resulted in their own financial situation deteriorating.

The aim of the study was to identify factors correlating with the self-rated quality of life of elderly inhabitants of cities applying for social help.

### Methods selection of the studied group

**Figure 1:**
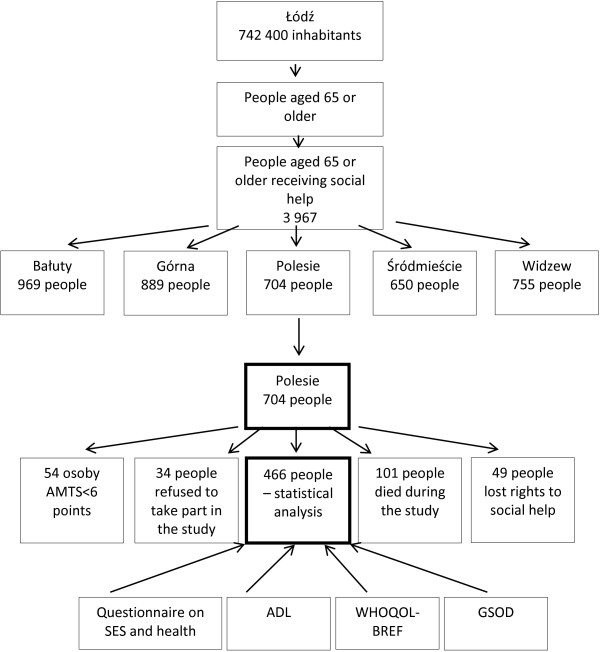
The scheme of the authors’ own study.

The study was conducted in Łódź – the third largest Polish city, between September 2011 and February 2012 in a group of people applying for help in the Municipal Social Welfare Centre in one city district. The welfare centre was randomly selected from five centres (Figure 1).

At the end of 2010, the population of this district was 143,400 people and inhabitants aged 65 or older made up 16.8% of the city population. The feminization rate in the studied subpopulation was 121.7. In 2010 5,336 people were eligible for social allowances in the studied centre. People aged 65 or older made up 23.7% of the total number of people who were eligible to seek social help (574 females and 130 males). The inclusion criteria were age 65 years and the mental ability to answer the interview questionnaire. In order to evaluate mental state, the authors used the Hodgkinson Abbreviated Mental Test Score. Fifty-four people were not qualified for further stages of the study due to their insufficient mental state. During the studied period, 101 respondents died. Each respondent was asked to give their written consent for such a study to be conducted. Thirty-four people refused to participate in the study. In the studied period 49 people lost their right to receive social help. Eventually, information on 466 respondents aged 65 or older was included in the statistical analysis.

It is worth mentioning that during the study period, 3,967 people, aged 65 or older, coming from the city of Łódź, became eligible to receive social help. Those who received help from the welfare centre included in the study made up 17.7% of elderly people. In Table 1 the authors compared the profile of the group included in the study, with refard to sex, age and type of received social help, with that of other elderly visitors to the Municipal Social Welfare Centre in Łódź. The authors did not observe significant differences with regards to the above variables. All those elderly people seeking help in the Municipal Social Welfare Centre in Łódź, as with those included in the study, were most often 80–84 years old. Females rather than males applied for social help; they made up 80.5% of the total number of social help seekers. The type of help was also similar; most often the people asked for nursing services. We can thus conclude that the randomly selected group was representative of the whole subpopulation aged 65 or older receiving social help in the city of Łódź.

**Table 1:** Comparison of the studied group with the whole population of elderly people applying for social help in Łódź

**Variable**		**Studied group**	**Population of applicants**	**The Pearson’s chi-square test**	**p**
		**n = 466**	**N = 3967**		
		**%**	**%**		
Sex	Males	22,1	19,5	1,966	p > 0,05
	Females	77,9	80,5		
Age	65–69	19,8	18,7	2,625	p > 0,05
	70–74	13,3	14,3		
	75–79	20,9	18,5		
	80–84	24,2	25,9		
	85+	21,8	22,6		
Kind of help	Financial	20,3	19,0	0,499	p > 0,05
	Nursing	79,7	81,0		

Source: the authors’ own calculations.

We obtained the approval of the Bioethics Committee of the Medical University in Lodz to carry out this study (decision RNN/109/12/KB of February 2, 2011).

### Methods and study tools

An interview questionnaire referring to the respondents’ health, financial, demographic and family situation was applied. The questionnaire consisted of 87 questions. It was divided into 5 parts which were preceded by the Abbreviated Mental Test Score. The test was the criterion for inclusion to the study. The first part of the questionnaire contained questions on personal data (age, sex, marital status, kind of work done, source of financial support). The second part referred to housing conditions, family relationships and also reasons for seeking social help and the form of received help; the third part referred to the financial situation of the respondents, measured with their income. The fourth part contained questions on health and quality of life of the subjects as well as their lifestyle. The authors included the Activity of Daily Living Scale (ADL) in the fourth part of the questionnaire. The last part presented the opinion of the social worker responsible for the respondent. Apart from these parts of the questionnaire the authors also used the Geriatric Depression Scale (GSOD) and the WHOQOL-BREF Questionnaire.

The complex character of the questionnaire used in this study allowed many variables correlating with self-rated quality of life of elderly people to be identified, evaluated with Question no. 1 of the WHOQOL-BREF Questionnaire. For the purposes of this article the authors selected the following variables for the statistical analysis: age, sex, marital status, education, kind of work done in the past, the number of people in the household, self-evaluation of financial income, health disorders and participation in family gatherings. Table 2 presents categories of the mentioned variables in the studied group.

**Table 2:** Characteristics of the studied group

**Variable**		**Males**		**Females**		**In total**	
		**n**	**%**	**n**	**%**	**N**	**%**
Sex		103	100.0	363	100.0	466	100.0
Age	65–69	41	40.2	50	14.0	91	19.8
	70–74	15	14.7	46	12.9	61	13.3
	75–79	22	21.6	74	20.7	96	20.9
	80–84	15	14.7	96	26.9	111	24.2
	85 and older	9	8.8	91	25.5	100	21.8
Marital status	Single Male/single female	15	14.6	43	11.8	58	12.4
	Married	10	9.7	9	2.5	19	4.1
	Widower/widow	45	43.7	268	73.9	313	67.2
	Divorced	33	32.0	43	11.8	76	16.3
Education	Elementary	42	40.8	200	55.1	242	51.9
	Vocational	18	17.5	19	5.2	37	7.9
	Secondary	34	33.0	119	32.8	153	32.9
	University	9	8.7	25	6.9	34	7.3
Work done in the past	Manual worker	84	81.6	239	65.8	323	69.3
	Desk-bound worker	19	18.4	124	34.2	143	30.7
Number of people in the household	One	91	88.4	334	92.0	425	91.2
	More than one	12	11.6	29	8.0	41	8.8
Sufficient income	Yes	7	6.8	25	6.9	32	6.9
	No	96	93.2	338	93.1	434	93.1
Health disorders	Headaches	71	68.9	312	85.9	383	82.2
	Pain in the spine and joints	34	33.0	252	69.4	286	61.4
	Pain in the chest	49	47.6	199	54.8	248	53.2
	Shortness of breath	63	61.2	215	59.2	278	59.7
	Heart palpitations	29	28.2	156	42.9	185	39.7
	Unstable blood pressure	51	49.5	189	52.1	240	51.5
	Tiredness	69	66.9	275	75.8	344	73.8
Participation in family gatherings	Every week	0	0.0	2	0.6	2	0.4
	Every month	2	1.9	9	2.5	11	2.4
	A few times a year	29	28.2	191	52.5	220	47.2
	Once a year	1	1.0	1	0.3	2	0.4
	Never	71	68.9	160	44.1	231	49.6
Self-rated quality of life	Good and very good	16	15.5	64	17.6	80	17.2
	Neither good nor bad	61	59.2	200	55.1	261	56.0
	Bad and very bad	26	25.3	99	27.3	125	26.8
Activity of daily living (ADL)	Fitness	70	67.9	229	63.2	299	64.2
	Moderate disability	27	26.2	98	26.9	125	26.8
	Disability	6	5.9	36	9.9	42	18.6
Geriatric scale of Depression (GSOD)	No depression	8	7.8	70	19.3	78	16.7
	Diagnosed/not diagnosed depression	31	30.1	88	24.2	119	25.5
	Possible depression	64	62.1	205	56.5	269	57.7

Source: the authors’ own calculations.

The WHOQOL-BREF Questionnaire is a study tool used in evaluation of the quality of life of healthy and sick people. The BREF version consists of 26 questions. In the first question the respondents were presented a set of answers: very good, good, neither good nor bad, bad, very bad. For the purposes of analysis the authors combined the categories “bad” with “very bad” and also “good” with “very good” because the variants “very good” and “very bad” were ticked by hardly any respondents (4 people and 1 person respectively). The authors also applied the ADL Scale, which is needed to evaluate the ability to get dressed without assistance, mantain personal hygiene, walk, use a toilet, eat, control the sphincter. For each activity the respondent could obtain three points. The subjects were asked to choose one option of the three given: completely independent, requiring assistance, reliant on other people’s help. The respondent could get a maximum of 6 points. The score 5 – 6 points meant complete independence, 3 – 4 points – moderate disability, 2 – 3 points – inability to live independently. The mental state of the respondents was assessed with the GSOD Scale. The respondent’s mental state was evaluated over a period of two weeks when asked four questions. The answers were: “Yes” or “No”. The more points the respondent received the more likely it was he suffered from depression. A score of 0 meant the person did not suffer from depression; a score of 1 meant that depression can be both confirmed or excluded. A score higher than 2 points meant the respondent suffered from depression.

The statistical relationship was analyzed at the significance level p < 0.5. The relationship between self-rated health and selected variables was analyzed with the use of single-factor regression and multiple regression.

### Statistical analysis

The authors analyzed the following study hypotheses:

1. The people with a better ADL and GSOD score have a better self-reported quality of life.

2. Not complaining of health disorders results in a more positive self-rated quality of life; thus, those who require nursing services are less likely to regard theirs as good.

3. Participating in family gatherings contributes to a higher self-rated quality of life.

The obtained data were entered into a Microsoft Excel database. The statistical significance level was p < 0.05. The authors used the following descriptive and analytical statistics measures: arithmetic mean, median, modal, ratios of the structure – percentage and fractions, which depended on the number of the studied groups analyzed according to the variables. The Pearson’s *x*^*2*^ test was used to compare the structure of the studied group with regards to sex, age and the form of received help and the whole subpopulation, aged 65 or older using social help all around the city.

The evaluation was made at a level of significance of p < 0.05. The authors applied single-factor and multiple logistic regression in order to evaluate the relationship between the self-rated quality of life of the studied respondents and selected variables. Two models of the single-factor logistic regression were created: I – good versus bad and II - neither good nor bad. Only one model identified by multiple logistic regression: good self-rated quality of life versus bad and neither good nor bad. The Statistica 9.0 Program (StatSoft, US) was used for statistical purposes.

## Results and discussion

### Results

The studied group consisted of 363 females (77.9%) and 103 males (22.1%). They were aged 65 – 100 years and the mean age was 79 ± 9.87 years. The majority of the respondents had elementary education (52.0% of the subjects). More than half the people were widowed (67.2%). When asked about the quality of their life, the respondents most often answered “neither good nor bad” (56.0%). 26.8% of the respondents claimed the quality of life is bad or very bad (25.3% males and 27.3% females), whereas 17.2% of the subjects said the quality was very good. Most of the subjects complained of health disorders. 82.2% suffered from frequent headaches, 73.8% - tiredness, 61.4% - pain in the spine and joints, 59.7% - shortness of breath, 53.2% - pain in the chest, 51.5% - unstable arterial blood pressure and 39.7% observed heart palpitations. The authors did not note statistically significant differences between the sex and particular health disorders. Only pain in the spine and joints were observed twice as often in females as in males. Table 2 presents these characteristics.

In order to evaluate the influence of the studied variables the authors used model I of the single-factor logistic regression. This model appeared to be better than model II. With regards to the single-factor logistic regression 12 variables selected for the purposes of this analysis turned out to contribute significantly to a better quality of life. They included: education, sufficient income, nursing services as form of received help, the score of the ADL Scale, no headaches, no pain in the chest or shortness of breath, stable blood pressure, no tiredness, no heart palpitations, participation in family gatherings, the score on the GSOD Scale which confirms the respondent does not suffer from depression.

University education increased the possibility of enjoying good life more than twice (OR = 2.31; p < 0.05). If the respondent’s income was sufficient enough for him or her to satisfy needs, the quality of life was 1.5 times higher (OR = 1.63; p < 0.05). The subjects who were provided with nursing services almost twice less frequently claimed that the quality of their life is good in comparison with those who were provided with financial help (OR = 0.45). It might be concluded that deteriorated health is the main reason for applying for nursing services. According to the ADL Scale, healthy people claimed that the quality of their life is more often than twice in comparison to disabled people (OR = 0.47) – p < 0.05. The fact that the respondents did not complain of heart palpitations, increased a chance for good life quality more than twice (OR = 2.32); similarly no everyday tiredness (OR = 2.08) and stable arterial blood pressure (OR = 1.79) increased a chance for good life quality almost twice. Those who did not feel headaches, pain in the chest or shortness of breath more often considered their life good than the respondents who complained of these mentioned disorders. The odds ratios were: 1.55; 1.51 and 1.51 respectively (p < 0.05; p < 0.01). A similar observation was made for participation in family reunions. Those who did not participate in such reunions claimed less frequently than twice that the quality of their life is good (OR = 0.52; p < 0.05). The score of the GSOD Scale appeared to be the most import ant factor and it greatly affected the person’s quality of life. The odds ratio for the studied respondents who obtained between 0 and 9.88 on the GSOD Scale demonstrated lack of depression. Such respondents almost ten times more frequently claimed that the quality of their life is good than those with the score >1, which means possible depression. Also in the case of those with the value 1, which means they might or not suffer from depression, the chances for good life increased 6 times (OR = 6.33). The error probability in both the cases was 0.001. The other studied variables did not significantly contribute to the quality of life. Some variables actually increased chances for a better life quality; however, the difference was statistically insignificant. It is worth pointing out that age was such a variable. Younger respondents considered their life better than older ones. Table 3 presents the evaluation of possible good and bad self-rated quality of life in the analyzed two models of the single-factor logistic regression.

**Table 3:** Evaluation of chances for good (Model I) and bad (Model II) self-rated quality of life of elderly people applying for social help - single-factor logistic regression

**Variable**		**Model I**			**Model II**		
		**OR**	**95% CI**	**p**	**OR**	**95% CI**	**p**
Sex	Females	1.16	0.64–2.212	p > 0.05	1.00	Reference group	
	Males	1.00	Reference group		1.11	0.67–1.84	p > 0.05
Age	65–69	1.42	0.65–3.09	p > 0.05	1.00	Reference group	
	70–74	1.63	0.70–3.80	p > 0.05	0.61	0.28–1.32	p > 0.05
	75–79	1.13	0.51–2.52	p > 0.05	1.14	0.62–2.10	p > 0.05
	80–84	1.83	0.89–3.77	p > 0.05	0.76	0.41–1.42	p > 0.05
	85 and older	1.00	Reference group		1.02	0.56–1.87	p > 0.05
Marital status	Single male/single female	0.58	0.17–2.00	p > 0.05	1.86	0.47–7.32	p > 0.05
	Widower/widow	0.58	0.20–1.69	p > 0.05	1.89	0.54–6.69	p > 0.05
	Divorced	0.47	0.14–1.59	p > 0.05	2.61	0.69–9.86	p > 0.05
	Married	1.00	Reference group		1.00	Reference group	
Education	University	2.31	1.02–5.23	*p < 0.05*	1.00	Reference group	
	Secondary	1.24	0.72–2.13	p > 0.05	1.23	0.49–3.07	p > 0.05
	Vocational	0.87	0.32–2.37	p > 0.05	0.90	0.28–2.91	p > 0.05
	Elementary	1.00	Reference group		1.69	0.71–4.09	p > 0.05
Work done in the past	Manual worker	1.21	0.72–2.03	p > 0.05	1.00	Reference group	
	Desk-bound worker	1.00	Reference group		1.33	0.84–2.09	p > 0.05
Sufficient income	Yes	1.63	1.00–2.66	*p < 0.05*	1.03	Reference group	
	No	1.00	Reference group		1.00	0.68–1.56	p > 0.05
Number of people in the household	One	0.99	0.51–1.93	p > 0.05	1.00	Reference group	
	More than one	1.00	Reference group		0.87	0.43–1.78	p > 0.05
Kind of help	Financial	0.45	0.23–0.85	*p < 0.05*	1.23	0.49–3.07	p > 0.05
	Nursing	1.00	Reference group		1.00	Reference group	
Headaches	No	1.55	0.33–0.93	*p < 0.05*	1.00	Reference group	
	Yes	1.00	Reference group		0.83	0.55–1.26	p > 0.05
Pain in the spine and joints	No	0.78	0.47–1.29	p > 0.05	1.00	Reference group	
	Yes	1.00	Reference group		0.91	0.60–1.38	p > 0.05
Pain in the chest	No	1.51	0.31–0.84	*p < 0.01*	1.00	Reference group	
	Yes	1.00	Reference group		1.28	0.85–1.93	p > 0.05
Shortness of breath	No	1.51	0.31–0.84	*p < 0.01*	1.00	Reference group	
	Yes	1.00	Reference group		1.28	0.85–1.93	p > 0.05
Heart palpitations	No	2.32	1.15–4.70	*p < 0.05*	1.00	Reference group	
	Yes	1.00	Reference group		0.95	0.58–1.55	p > 0.05
Unstable blood pressure	No	1.79	1.06–3.03	*p < 0.05*	1.00	Reference group	
	Yes	1.00	Reference group		1.19	0.79–1.81	p > 0.05
Tiredness	No	1.79	1.06–3.03	*p < 0.05*	1.00	Reference group	
	Yes	1.00	Reference group		0.86	0.57–1.29	p > 0.05
Participation in family gatherings	No	0.52	0.36–0.86	*p < 0.05*	1.17	Reference group	
	Yes	1.00	Reference group		1.00	0.77–1.77	p > 0.05
ADL	Fitness	0.47	0.27–0.84	*p < 0.05*	1.00	Reference group	
	Moderate disability	0.61	0.29–1.29	p > 0.05	1.45	0.86–2.44	p > 0.05
	Disability	1.00	Reference group		0.56	0.24–1.30	p > 0.05
GSOD	No depression	9.88	4.99–19.6	*p < 0.001*	1.00	Reference group	
	Diagnosed/not diagnosed depression	6.33	3.32–12.1	*p < 0.001*	0.50	0.21–1.24	p > 0.05
	Possible depression	1.00	Reference group		3.41	1.75–6.63	*p < 0.001*

Source: the authors’ own calculations.

The authors used the multi-factor logistic regression in order to simultaneously evaluate the influence of factors, which, in the single-factor logistic regression, made the respondents from the Municipal Social Welfare Centre perceive their life positively. With regards to the multi-factor logistic regression there were 3 variables (of 12) which significantly contributed to a good quality of life. They included: education (p < 0.05), participation in family reunions (p < 0.01) and the score on the GSOD Scale (p < 0.001). It turned out that in the group of respondents with university education, rather than with elementary education, the chance for a positive quality of life increased 3 times (OR = 3.07). Those participating in family reunions more frequently than twice claimed their life was good than those who avoided such reunions (OR = 2.26). In the multi-factor logistic analysis, similarly to the single-factor logistic regression, the score on the GSOD Scale was the most influential. It was confirmed that the respondents whose score on the scale was 0 (no depression) claimed their life is better more often than ten times in comparison with those whose score was >1 (OR = 10.2). With regards to the subjects with the score 1, the chances for good life increased 7 times (OR = 7.02). Table 4 demonstrates the possibility of good self-rated quality of life of the respondents in the multi-factor logistic analysis.

**Table 4:** Evaluation of chances for good and bad self-rated quality of life of elderly people applying for social help – multi-factor logistic regression

**Variable**		**Good vs. bad, neither good nor bad**		
		**OR**	**95% CI**	**p**
Education	University	3.07	1.21–7.80	*p < 0.05*
	Secondary	1.27	0.68–2.37	p > 0.05
	Vocational	0.91	0.30–2.80	p > 0.05
	Elementary	1.00	Reference group	
Sufficient income	Yes	1.42	0.81–2.50	p > 0.05
	No	1.00	Reference group	
Kind of help	Financial	0.89	0.28–2.81	p > 0.05
	Nursing	1.00	Reference group	
Headaches	No	1.59	0.83–2.96	p > 0.05
	Yes	1.00	Reference group	
Pain in the chest	No	1.04	0.18–6.06	p > 0.05
	Yes	1.00	Reference group	
Shortness of breath	No	1.04	0.18–6.06	p > 0.05
	Yes	1.00	Reference group	
Heart palpitations	No	0.67	0.18–2.41	p > 0.05
	Yes	1.00	Reference group	
Unstable blood pressure	No	3.34	1.08–13.3	p > 0.05
	Yes	1.00	Reference group	
Tiredness	No	2.22	0.33–14.8	p > 0.05
	Yes	1.00	Reference group	
Participation in family gatherings	Yes	2.26	2.24–4.11	*p < 0.01*
	No	1.00	Reference group	
ADL	Fitness	0.56	0.23–1.35	p > 0.05
	Moderate disability	0.63	0.26–1.49	p > 0.05
	Disability	1.00	Reference group	
GSOD	No depression	10.2	4.93–21.1	*p < 0.001*
	Diagnosed/not diagnosed depression	7.02	3.51–14.0	*p < 0.001*
	Possible depression	1.00	Reference group	

Source: the authors’ own calculations.

## Discussion

A systematic increase in the percentage of elderly people, i.e., aged 65 or older, in Poland but also in Europe and the world, is mentioned in many publications [23]–[29]. While analyzing old age we do not consider only the years of life the person lived, but also how they were lived [30,31]. Lifestyle and health behaviours greatly affect the person’s health. Here we should mention a physical, mental and educational activity, a proper diet, relax and avoiding alcohol, nicotine etc. These factors are considered components of prophylaxis of premature ageing [32,33]. Many factors – social, biological and mental ones - contribute to the quality of life [34]–[42]. The authors of the study confirmed that the quality of life of the elderly depends on many elements: health, financial situation and family relationships. In professional literature there are a lot of studies on this problem so results of these might be sometimes extremely different. What makes this study innovative and original is the fact that it has information on the aspect of using social help by elderly people. This aspect has been hardly ever discussed in other studies.

Studies on the quality of life of the elderly (residents of nursing homes and participants of classes held by third-age universities) conducted in the Podkarpackie voivodeship, confirmed that social and demographic factors affected the quality of life of the respondents. These factors particularly included: sex, marital status and health. Age turned out to be less important. Other publications did not clearly show to what extent age is important. Age on its own is not a determining factor. However, in a combination with other variables (diseases, mental and physical disabilities) it can significantly decrease the quality of life [43]. The authors of this study made a similar observation. Although they did not note a statistically significant relationship between age and the quality of life, the calculated odds ratios confirmed that the possibility of enjoying good life is greater for younger respondents. A relationship between health and self-rated quality of life was also confirmed. The authors proved that in the respondents who did not suffer from disorders such as: headaches, pain in the chest, heart palpitations, unstable blood pressure, shortness of breath, tiredness the chances for a good quality of life increased more than twice. Studies conducted in Finland by Noro et al. [44] confirmed that marital status was also a significant factor. People who remain single or who have lost their spouse, often lack self-confidence. They have to face up to a new reality which is often connected with deterioration of their financial situation. This problem is accompanied by loneliness and isolation. Their social contacts become less and less frequent, which makes them feel even more lonely. Often meetings with friends or family motivate to activity and increase self-rated quality of life. The feeling that the elderly person is someone important makes him or her feel needed and helps to cope with personal failures. The author did not observe a direct relationship between the marital status and the quality of life but they observed a relationship between the quality of life and family relationships. It can be somehow identified with a positive effect if the person is not socially excluded. People actively involved in family gatherings were more likely to enjoy good life that those who did not participate in such meetings. Tseng and Wang conducted a study on 161 residents of 10 nursing homes in Taiwan and concluded that the quality of life also depends on education. Better education was connected with leading more healthy lifestyle [45]. The authors of this study also confirmed a relationship between the level of education and the quality of life. Depression is becoming more and more serious factor in a subpopulation of the elderly. This study demonstrates this trend, too. The BREF version of the GSOD Scale was applied as a study tool to diagnose depression. According to the results 62.1% of males and 56.5% of females suffered from depression. This disease significantly decreases the quality of life of the respondents. Depression which is untreated negatively affects the quality of life of the person and what is more, worsens other problems, which eventually might lead to death. It was confirmed that people who have had episodes of myocardial infarct or stroke and now have depression, are more threatened with death. According to American Health and Retirement Study both males and females are likely to develop depression in older age [46]. In 2006 – 2007 the Clinic of Geriatrics and internal diseases in Cracow conducted a study on the influence of depression on the quality of life of patients aged 80 or older with ischemic heart disease. Thanks to the analysis of the 15-score GSOD Scale the researchers evaluated the intensity of depression symptoms. They observed progressing depression in 30.9% patients. The developing depression was accompanied by problems with walking, taking care of themselves and, like in this study, appearing somatic symptoms [43]. Rogers et al. examined a group of 1,024 patients with coronary disease and observed depression in 20% of them. Such people significantly more often complained of a deteriorated quality of life, physical disabilities and their overall health was worse [47]. Wells et al. noted that depression makes everyday activities more difficult to perform [48]. Studies conducted in Taiwan in 1994 – 2004 showed that people aged 65 or older are more likely to suffer from depression than younger ones. In 1994 the percentage of people in this age group was 16.3%, but 10 years later it was 29.9% [49]. Moreover, in people with chronic diseases, depression symptoms are observed more frequently. This fact was also confirmed by the authors of this study.

Ageing populations are affected by important economic, social and medical problems. The WHO is aware of these problems and it points out the necessity to initiate activities which would make elderly people constantly involved in social life and which prevent this subpopulation against being affected by negative health, mental and social tendencies. Any initiatives should be exemplified by providing care for the elderly. Our objectives should not only aim at extending human life but also concentrating on improving the quality of life in all its aspects [50,51]. Many factors, such as: diseases, lifestyle, individual ageing processes as well as social, psychological and environmental factors contribute to the person’s fitness. Over years people are less and less independent and they need some other people to help them [52,53]. The authors confirmed that the kind of received social help significantly affected the quality of life of the person. The respondents who applied for nursing services negatively evaluated their quality of life, which is definitely related to their bad health. Thus, we can conclude that a demand for nursing care provided by welfare centres will be growing. The ability to provide self-care and being fit will allow in the elderly to remain independent in satisfying their needs which include: walking, eating, controlling physiological processes and keeping personal hygiene [54]–[56].

Demographic forecasts for coming years clearly confirm that ageing trends will be more and more visible. This will have biological, medical, economic and social implications [57]–[59]. Elderly patients need professional care. People responsible for modern social and health policy should implement initiatives which will improve both the health and quality of life of ageing populations [60,61].

## Conclusions

In the light of problem of ageing populations the issue of the quality of life of the elderly has become highly important. The analysis of factors determining the quality of life of people who apply for help to the Municipal Social Welfare Centre in Łódź allowed for drawing the following conclusions:

1. The quality of life of elderly people depends on many factors – mainly on physical and mental health, and which is directly connected with this, being provided with nursing care. Other factors include education, income and family relationships.

2. Symptoms of depression most significantly contributed to a negative self-rated quality of life.

3. Identifying conditions of the quality of life of elderly people might help implement initiatives aiming at the improvement of the quality of life and, as a consequence, decrease a demand for social help, i.e. nursing services.

## Abbreviations

ADL: Activity of daily living; AMTS: Abbreviated mental test score; GSOD: Geriatric depression scale; HRQOL: Health related quality of life; MOPS: Municipal social welfare centre; QOL: Quality of life; SES: Social and economic situation; WHO: World Health Organization.

## Competing interests

The authors declare no competing interest.

## Authors’ contributions

Marek B – selecting literature, preparing and editing the manuscript; Monika B – preparing the methodology of the study, collecting data, the analysis of results and preparing the manuscript; IM-B – preparing the idea and methodology of the study, monitoring the completion of the study, preparing the manuscript. All the authors read and adopted the manuscript.

## References

[B1] Mohan R Beydoun H Beydoun M Schellhammer P Self-rated health as a tool estimating health-adjusted life expectancy among patients newly diagnosed with localized prostate cancer: a preliminary study Qual Life Res 2011 20 5 713 721 10.1007/s11136-010-9805-3 21132389 PMC3066264

[B2] Baker R Baleman I Donaldson C Weighting and valuing quality-adjusted life-years using stated preference methods: preliminary results from the Social Value of a QUALY Project Health Technol Assess 2010 14 7 7 18 10.3310/hta14270 20525460

[B3] Porta M A Dictionary of Epidemiology 2008 USA: Oxford University Press

[B4] Karnofsky DA Burchenal JH MacLeod CM The Clinical Evaluation of Chemotherapeutic Agents in Cancer Evaluation of Chemotherapeutic Agents 1949 New York: Columbia University Press 196

[B5] World Health Organisation Preamble to the Constitution of the World Health Organization as adopted by the International Health Conference, Volume 2 1948 New York: WHO 100 19–22 June, 1946; signed on 22 July 1946 by the representatives of 61 States

[B6] Austin PC A comparison of methods of analyzing health-related quality-of-life measures Value Health 2002 5 4 329 337 10.1046/j.1524-4733.2002.54128.x 12102695

[B7] Reijula J Rosendahl T Reijula K Roilas P Roilas H Sepponen R A new method to assess perceived well-being among elderly people – a feasibility study BMC Geriatr 2009 9 55 62 10.1186/1471-2318-9-55 19958553 PMC2791757

[B8] van Soest-Portyliet MC van der Steen JT Zimmerman S Cohen LW Reed D Archterberg WP Ribbe MW de Vet HC Psychometric properties of instruments to measure the quality of end-of-life care and dying for long-term care residents with dementia Qual Life Res 2012 21 671 684 10.1007/s11136-011-9978-4 21814875 PMC3323818

[B9] Bowden A Fox-Rushby J Nyandieka L Wanjau J Methods for pre-testing and piloting survey questions: illustrations from the KENQOL survey of health-related quality of life Health Policy Plann 2002 17 3 322 330 10.1093/heapol/17.3.322 12135999

[B10] Chan S Jia S Chiu H Chien W Hu Y Lam L Subjective health-related quality of life of Chinese older persons with depression in Shanghai and Hong Kong: relationship to clinical factors level of functioning and social support Int J Geriatr Psychiatry 2009 24 355 362 10.1002/gps.2129 18773498

[B11] Davis JC Marra C Najafzadeh M Liu-Ambrose T The independent contribution of executive functions to health related quality of life in older women BMC Geriatr 2010 10 16 24 10.1186/1471-2318-10-16 20359355 PMC2867806

[B12] Debpuur C Welaga P Wak G Hodgson A Self-reported health and functional limitations among older people in the Kassena-Nankana District, Ghana Global Health Action 2010 2 54 63 10.3402/gha.v3i0.2151 PMC2957305 20963186

[B13] Roe B Beynon C Pickering L Duffy P Experiences of drug use and ageing, health, quality of life, relationship and service implications J Adv Nurs 2010 66 9 1968 1979 20626477 10.1111/j.1365-2648.2010.05378.x PMC2984546

[B14] Breeze E Sloggett A Fletcher A Socioeconomic and demographic predictors of mortality and institutional residence among middle aged and older people: results from the longitudinal study J Epidemiol Community 1999 53 765 774 10.1136/jech.53.12.765 PMC1756816 10656085

[B15] Tidermark J Zethraeus N Svensson O Törnkvist H Ponzer S Femoral neck fracture in the elderly: Functional outcome and quality of life according to EuroQOL Qual Life Res 2002 11 473 481 10.1023/A:1015632114068 12113394

[B16] Maniecka-Bryła I Drygas W Bryła M Dziankowska-Zaborszczyk E Determinants of self-rated health among the elderly living in a big city environment Pol J Env Stud 2011 20 691 699

[B17] Pikala M Maniecka Bryla I Years of life lost due to malignant neoplasms characterized by the highest mortality rate Arch Med Scidoi:10.5114/aoms.2013.36237 10.5114/aoms.2013.36237 PMC4223125 25395953

[B18] Borg C Hallberg IR Blomgvist K Life satisfaction among older people (65+) with reduced self-care capacity: the relationship to social, health and financial aspects J Clin Nurs 2006 15 607 618 10.1111/j.1365-2702.2006.01375.x 16629970

[B19] Iwarsson S Isacsson A Quality of life in an elderly population an example exploring interrelationship among subjective well-being, ADL dependence, and housing accessibility Arch Gerontol Geriat 1997 26 71 83 10.1016/S0167-4943(97)00034-4 18653127

[B20] Duru G Auray JP Béresniak A Lamure M Paine A Nicoloyannis N Limitations of the methods used for calculating Quality-adjusted life-year values Pharmacoeconomics 2002 20 7 463 473 10.2165/00019053-200220070-00004 12093302

[B21] Central Statistical Office Trwanie życia w Polsce 2011 2012 Warszawa: Central Statistical Office in Polish

[B22] Maniecka-Bryła I Pikala M Bryła M Health inequalities among rural and urban inhabitants of Łódź province, Poland Ann Agr Env Med 2012 19 723 731 23311797

[B23] Bowling A Aspirations for older age in the 21^st^ century. What is successful aging Int J Aging Hum Dev 2007 64 3 263 297 10.2190/L0K1-87W4-9R01-7127 17503689

[B24] Britton A Shipley M Singh-Manoux A Marmot MG Successful aging: the contribution of early-life and midlife risk factors J Am Geriatr Soc 2008 56 6 1098 1103 10.1111/j.1532-5415.2008.01740.x 18482302 PMC2696176

[B25] Zielińska-Więczkowska H Kędziora-Kornatowska K Quality of ageing and old age in personal opinions of the members of University of the Third Age Geront Pol 2009 17 3 137 142 (in Polish)

[B26] Jagger C Gillies C Moscone F Cambois E van Oyen H Nusselder W Robine JM EHLEIS team Inequalities in healthy life years in the 25 countries of the European Union in 2005: across-national meta-regression analysis Lancet 2008 372 12 2124 2131 19010526 10.1016/S0140-6736(08)61594-9

[B27] Jeste DV Feeling fine at a hundred and three Secrets success aging Am J Prev Med 2005 28 3 323 324 10.1016/j.amepre.2004.12.015 15766623

[B28] Rantakokko M Iwarsson S Kauppinen M Leinonen R Heikkinen E Rantanen T Quality of life and barriers in the urban outdoor environment in old age J Am Geriatr Soc 2010 58 2154 2159 10.1111/j.1532-5415.2010.03143.x 21054297

[B29] Kyobutungi C Egondi T Ezeh A The health and well-being older people in Nairobis slums Global Health Action 2010 2 45 53 10.3402/gha.v3i0.2138 PMC2957141 20959873

[B30] Evans E Gately C Huxley P Smith A Banerjee S Assessment of quality of life in later life: development and validation of the QuiLL Qual Life Res 2005 14 1291 3000 10.1007/s11136-004-5532-y 16047504

[B31] Gómez-Olivé FX Thorogood M Clarc BD Kahn K Tollman SM Assesing health and well-being among older people in rural South Africa Global Health Action 2010 2 23 35 10.3402/gha.v3i0.2126 PMC2957314 20963188

[B32] Baumann K Quality of life in old age — theoretical discourse Geront Pol 2006 14 4 165 171 (in Polish)

[B33] Benyamini Y Blumstein T Lusky A Modan B Gender differences in the self-rated health-mortality association: is it poor self-rated health that predicts mortality or excellent self-rated that predicts survival? Gerontologist 2003 43 3 396 405 10.1093/geront/43.3.396 12810904

[B34] Hughes RA Addington-Hall JM Aspinal F Developing methods to improve the quality of end-of-life care J Interprof Care 2004 18 2 200 201 10.1080/13561820410001686963 15203679

[B35] Johnson JA Coons SJ Comparison of the EQ-5D and SF-12 in an adult US sample Qual Life Res 1998 7 155 166 10.1023/A:1008809610703 9523497

[B36] Al-Janabi H Flynn TN Coast J Development of a self-report measure of capability wellbeing for adults: the ICECAP-A Qual Life Res 2012 21 167 176 10.1007/s11136-011-9927-2 21598064 PMC3254872

[B37] Alma MA van Dermei SF Groothoff JW Determinants of social participation of visually impaired older adults Qual Life Res 2012 21 87 97 10.1007/s11136-011-9931-6 21633880 PMC3254864

[B38] Clench-Aas J Nes RB Dalgard OS Aarϕ LE Dimensionality and measurement invariance in the Satisfaction with Life Scale in Norway Qual Life Res 2011 20 1307 1317 10.1007/s11136-011-9859-x 21308414 PMC3178031

[B39] Loyland B Miaskowski C Paul SM Dahl E Rustoen T The relationship between chronic pain and health-related quality of life in long-term social assistance recipients in Norway Qual Life Res 2010 19 1457 1465 10.1007/s11136-010-9707-4 20652418 PMC2977061

[B40] Garster NC Palta M Sweitzer NK Measuring health-related quality of life in population-based studies of coronary heart disease: comparing six generic indexes and a disease-specific proxy score Qual Life Res 2009 18 1239 1247 10.1007/s11136-009-9533-8 19760103 PMC2759459

[B41] Maida CA Marcus M Spolsky VW Wang Y Liu H Socio-behavioral predictors of self-reported oral health-related quality of life Qual Life Res 2012 13 78 87 10.1007/s11136-012-0173-z PMC9637395 22528238

[B42] Sposito G D’Elboux-Diogo MJ Cintra F Neri AL Guariento ME Relationship between subjective well-being and the functionality of elderly outpatients Brasilian J Phys Ther 2010 14 1 81 89 10.1590/S1413-35552010000100013 20414566

[B43] Grzegorczyk J Kwolek A Bazarnik K Szeliga E Wolan A Jakość życia osób mieszkających w domach pomocy społecznej i słuchaczy uniwersytetu trzeciego wieku Przegl Med Uniw Rzesz 2007 5 3 225 233 (in Polish)

[B44] Noro A Aro S Health-related quality of life among the least dependent institutional elderly compared with the non-institutional elderly population Qual Life Res 1996 5 3 355 366 10.1007/BF00433920 8763804

[B45] Tseng SZ Wang RH Quality of life and related factors among elderly nursing home residents in Southern Taiwan Public Health Nurs 2001 18 5 304 311 10.1046/j.1525-1446.2001.00304.x 11559413

[B46] Ayotte BJ Physical Health and Depression: A Dyadic Study of Chronic Health Conditions and Depressive Symptomatology in Older Adult Couples J Gerontol B Psychol Sci Soc Sci 2010 66B 1 438 448 20498455 10.1093/geronb/gbq033 PMC2883871

[B47] Rogers MP White K Warshaw MG Yonkers KA Rodriguez-Villa F Chane G Keller MB Kreis IA Chiu HC Prevalence of medical illness in patients with anxiety disorders Int J Psychiatry Med 1994 24 1 83 96 10.2190/TXM9-EVX8-Q4WT-G03J 8077085

[B48] Wells KB Burnam MA Rogers W The course of depression in adult outpatients Results from the Medical Outcomes Study Arch Gen Psychiatry 1992 49 10 788 794 10.1001/archpsyc.1992.01820100032007 1417431

[B49] Chen CM Mullan J Griffiths D Trajectories of depression and their relationship with health status and social service use Arch Gerontol Geriat 2011 53 2 118 124 10.1016/j.archger.2010.07.006 20810178

[B50] Lewandowska A Olchowik G Expectations of nursing homes pensioners Wellness in different phases of life 2008 Lublin: Wydawnictwo Neuro Centrum 115 119

[B51] Abas MA Punpuing S Jirapramupitak T Tangchon K Leese M Psychological wellbeing, physical impairments and rural aging in a developing country setting Health Qual Life Outcomes 2009 7 66 http://dx.doi.org/10.1186/1477-7525-7-66 10.1186/1477-7525-7-66 PMC2723081 19607711

[B52] Al Windi A The relations between symptoms, somatic and psychiatric conditions, life satisfaction and perceived health. A primary care based study Health Qual Life Outcomes 2005 3 28 http://dx.doi.org/1477-7525-3-28 10.1186/1477-7525-3-28 PMC1131915 15857513

[B53] Central Statistical Office Sytuacja demograficzna Polski. Raport 2010–2011 2012 Warszawa: Central Statistical Office in Polish

[B54] Sawatzky R Liu Ambrose T Miller WC Marra CA Physical activity as a mediator of the impact of chronic conditions on quality of life in older adults Health Qual Life Outcomes 2007 68 http://dx.doi.org/1477-7525-5-68 10.1186/1477-7525-5-68 PMC2246116 18093310

[B55] Commission of the European Communities The 2012 Ageing Report 2012 25 69 COM

[B56] Wejnert B Wachowiak A Problematyka subiektywnej i obiektywnej oceny jakości życia w badaniach amerykańskich Jak żyć 2001 Poznań: Wydawnictwo Fundacji Humaniora 41 60 in Polish

[B57] Fleury MJ Grenier G Bamvita JM Tremblay J Schmitz N Caron J Predictors of quality of life in a longitudinal study of users with severe mental disorders Health Qual Life Outcomes 2013 92 http://dx.doi.org/10.1186/1477-7525-11-92 10.1186/1477-7525-11-92 PMC3681595 23758682

[B58] Maniecka-Bryła I Gajewska O Burzyńska M Bryła M Factors associated with self-rated health (SRH) of a University of the Third Age (U3A) class participants Arch Gerontol Geriat 2013 57 156 161 10.1016/j.archger.2013.03.006 23578848

[B59] Commission of the European Communities, COM The demographic future of Europe – from challenge to opportune. Main European demographic trends and data. Projections – EU-25 2006 Brussels: European Commission

[B60] Śmigiel J Poczucie jakości życia a aktywność osób w starszym wieku Geront Pol 1997 5 2 21 29 (in Polish)

[B61] Evans M Khunti K Mamdani M Galbo Jørgensen CB Gundgaard J Bøgelund M Harris S Health-related quality of life associated with daytime and nocturnal hypoglycaemic events: a time trade-off survey in five countries Health Qual Life Outcomes 2013 90 http://dx.doi.org/10.1186/1477-7525-11-90 10.1186/1477-7525-11-90 PMC3679729 23731777

